# Supplementary material for: Time-reversal symmetry breaking hidden order in Sr2(Ir,Rh)O4
Source: Nat Commun. 2017 Apr 24;8:15119. doi: 10.1038/ncomms15119 (PMC5413971; doi:10.1038/ncomms15119)
Supplement: Supplementary Information — Supplementary Figures, Supplementary Notes and Supplementary References [file ncomms15119-s1.pdf]

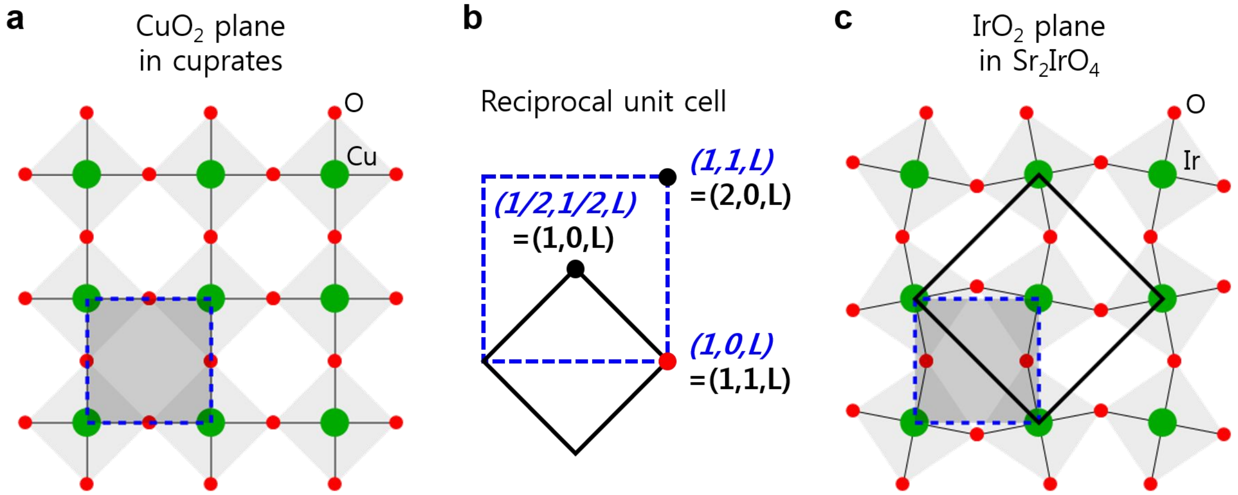

Supplementary Figure 1: **MO$_2$ plane in cuprates (M=Cu) and in Sr$_2$IrO$_4$ (M=Ir).** (a) In cuprates, the CuO_2 plane is typically a weakly distorted square lattice that one generally represents as a regular square lattice, so it could be described by the minimal unit cell (dashed blue). (c) In contrast, in iridates, due to a large in-plane rotation of the IrO_6 octahedra, the corresponding unit cell is doubled and rotated by 45° (black). (b) Planar reciprocal space where a few interesting **Q**-positions are compared in both systems (written in italic blue for cuprates and in black for iridates).

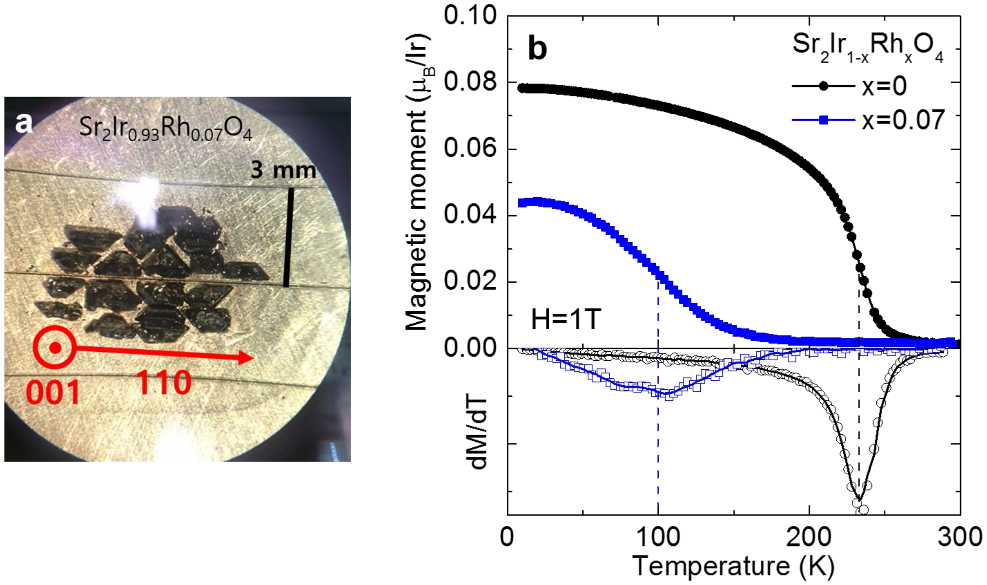

Supplementary Figure 2: **Coaligned single crystals and measured magnetic moments** (a) Total 18 single crystals of the 7% Rh-doped sample were coaligned on a thin Al plate. Two major crystallographic directions are denoted: $(1, 1, 0)$ and $(0, 0, 1)$. (b) Ferromagnetic moments deduced from magnetization measurements at $H = 1$ T. By substituting Rh for Ir, the magnetic transition temperature and the saturated moment are rapidly suppressed.

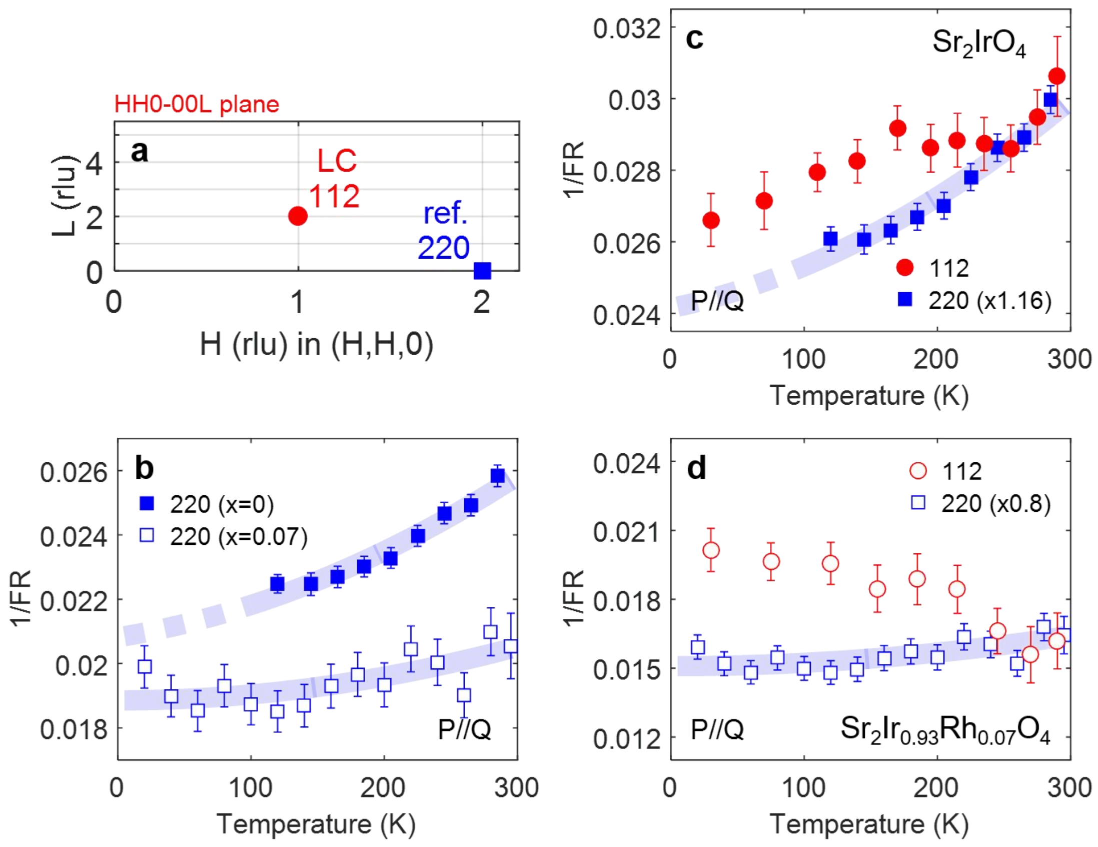

Supplementary Figure 3: **Comparison between the $(1,1,2)$ and the reference $(2,2,0)$ Bragg peaks.** (a) (H,H,L) scattering plane where the $(1,1,2)$ (red circle) and $(2,2,0)$ (blue square) Bragg peaks positions are underlined. (b) 1/FR measured at the reference postion (2,2,0) for both pure (full squares) and doped (empty squares) samples, showing a small drift of $1/FR_0$ in temperature. (c,d) The relative deviation of 1/FR at $(1,1,2)$ from the scaled $(2,2,0)$ for both systems. Vertical error bars are statistical errors (1 standard deviation).

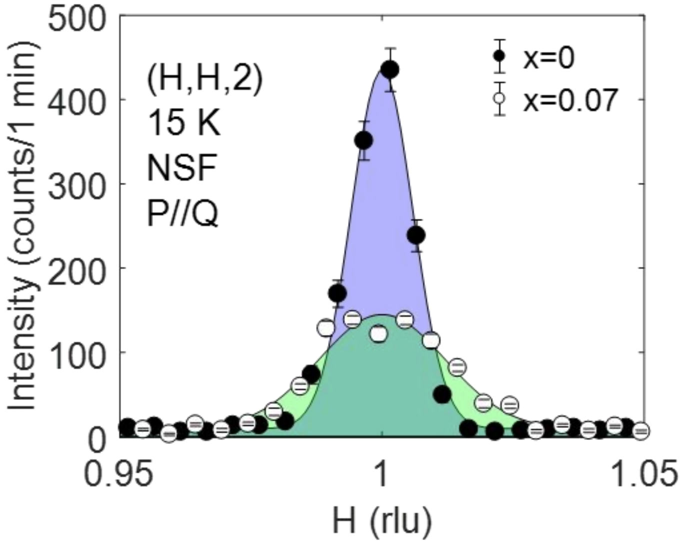

Supplementary Figure 4: H**-scan of the** $(1, 1, 2)$ **Bragg peak in the NSF channel.** The integrated intensity for the NSF channel at $(1, 1, 2)$ in the doped sample is about 1.6 times smaller than one in the pure sample. Vertical error bars are statistical errors (1 standard deviation).

Supplementary Note 1

Reciprocal space comparison with cuprates

The Sr_2IrO_4 exhibits a very similar in-plane structure compared to superconducting cuprates. In cuprates, corner-shared CuO_6 octahedra or CuO_5 pyramids make a (nearly) square lattice of CuO_2 plaquettes on the basal ab-plane. On the other hand in iridates, the IrO_6 octahedra are also corner-shared but rotated by $\theta \sim 11^\circ$ in the basal plane. Due to this in-plane rotation, the unit cell for iridates is doubled and rotated by 45° as shown in Supplementary Fig. 1. Despite a difference between the spin $S = 1/2$ of Cu^{2+} and pseudo-spin $J = 1/2$ of Ir^{4+} states, the antiferromagnetic (AFM) structure is also almost the same in both systems, so the magnetic Bragg conditions are also similar. However, under the 45° unit-cell transformation, actual (H, K, L) Miller indices are different. In the center of Supplementary Fig. 1, we compare a few interesting Bragg **Q**-positions in the planar reciprocal HK-plane for iridates (black) and cuprates (blue). For instance, the $(1/2, 1/2, L)$ for the AFM order in cuprates is transformed to $(1, 0, L)$ or $(0, 1, L)$ for iridates, and the $(1, 0, L)$ in cuprates, where the loop-current (LC) order has been reported [1,2], corresponds to $(1, 1, L)$ for iridates.

Supplementary Note 2

Analysis for the flipping ratio

According to the (co-planar) loop current (LC) model, the magnetic scattering intensity should be observed at Bragg reflections $(1, 1, 2 + 4n)$, on top of the nuclear scattering. As the LC phase respects the translation symmetry of the lattice, it corresponds to an intra-unit-cell magnetic order. Thus, the detection of such a magnetic order critically depends on the ability to disentangle nuclear and magnetic scatterings. This difficulty can be overcomed by using polarized neutron scattering technique [1,2]. Once the neutron spin polarization $\mathbf{P}$ is set parallel to the transferred momentum, $\mathbf{P} \parallel \mathbf{Q}$, the magnetic scattering purely appears in spin flip (SF) channel and the nuclear one in the non-spin-flip (NSF) channel. In principle, probing separately the SF and NSF scattering channels allows one to determine the magnetic and nuclear scatterings. However, polarized neutron experiments are always limited by the quality of the neutron spin polarization, which is given by FR, that should go to infinity for a perfectly polarized neutron beam. In practice, FR is finite and a fraction $1/\mathrm{FR}$ of the nuclear (*i.e.* NSF) scattering goes into the SF channel: it is referred to as the polarization leakage that determines a corresponding bare flipping ratio FR_0. Another important point is to determine possible temperature dependence of the bare flipping ratio $\mathrm{FR}_0(\mathrm{T})$. Indeed, when changing the temperature, very tiny changes of the experimental set-up may occur[2], producing a continuous drift of $\mathrm{FR}_0(\mathrm{T})$ that needs to be calibrated.

In order to reveal a small magnetic signal on top of the nuclear polarization leakage in the SF

channel, one has to pick up the weakest nuclear Bragg peaks (with the appropriate symmetry for the LC phase) to get the better signal-to-background ratio. In addition, the magnetic form factor is generally suppressed at a high momentum transfer. Considering these constraints, the $(1,1,2)$ reflection has been studied, which has the lowest $|\mathbf{Q}|$ of the allowed nuclear Bragg peaks $(1,1,2{+}4n)$ for the LC phase. Further, in order to determine the temperature dependence of the bare flipping ratio, $\mathrm{FR_0(T)}$, a non-magnetic reference Bragg peak is measured where the magnetic scattering is zero or small enough and where the spectrometer geometry is kept similar. Here, we chose the $(2,2,0)$ Bragg peak (see Supplementary Fig. 3a) as the reference because a magnetic signal is expected to be considerably reduced by the magnetic form factor and the spectrometer geometry is not changed much with respect to $\mathbf{Q} = (1,1,2)$. Using $\mathrm{FR_0(T)}$, obtained from the non-magnetic reference, one can then determine the intrinsic polarization leakage $\mathrm{I^0_{SF}} = \mathrm{I_{NSF}/FR_0(T)}$ and next extract from the SF intensity $\mathrm{I_{SF}}$ the true magnetic one at $\mathbf{Q} = (1,1,2)$, $\mathrm{I_{mag}} = \mathrm{I_{SF}} - \mathrm{I^0_{SF}}$.

For the pure sample, the $\mathrm{FR_0^{220}(T)}$ at the $(2,2,0)$ is ∼39 at 300 K and it increases up to ∼45 at 100 K, meaning that $1/\mathrm{FR_0(T)}$ (reported in Supplementary Fig. 3b) decreases upon cooling. That smooth decrease indicates no magnetic signal because one should observe a sudden increase of 1/FR in case of a magnetic order. The $\mathrm{FR_0^{220}(T)}$ for the doped sample is higher (∼50) (1/FR ~ 0.019 reported in Supplementary Fig. 3b) and much stable in temperature. That proves as well no magnetic signal at the $(2,2,0)$ peak in the doped sample. The observed temperature-dependent $1/\mathrm{FR^{220}(T)}$ can therefore be taken as the bare $1/\mathrm{FR_0(T)}$ for the polarization calibration.

In contrast, the $\mathrm{FR}^{112}(T)$ at the $(1,1,2)$ shows a clear change of slope around 200–250 K

as shown in Supplementary Fig. 3c,d. The $\mathrm{FR}^{112}(T)$ is $\sim$33–38 and $\sim$50–63 for the pure and doped sample, respectively. This main difference of the magnitude of FR originates from different experimental conditions such as sample mosaicity, the number of blades on the analyzer and optimization of the guide field. In order to estimate the bare $\mathrm{FR}_0^{112}(\mathrm{T})$ for the $(1,1,2)$, the $\mathrm{FR}_0^{220}(\mathrm{T})$ is scaled by a factor that gives the same value with the $\mathrm{FR}^{112}(T)$ at high temperature above 250 K. Then, as shown in Supplementary Fig. 3c-d, we clearly observe a departure of $\mathrm{FR}^{112}(T)$ from $\mathrm{FR}_0^{112}(\mathrm{T})$ at low temperature. Using this $\mathrm{FR}_0^{112}(\mathrm{T})$, the bare polarization leakage, $\mathrm{I}_{\mathrm{SF}}^0(\mathrm{T}) = \mathrm{I}_{\mathrm{NSF}}(\mathrm{T})/\mathrm{FR}_0^{112}(\mathrm{T})$ in the SF channel can be determined at $\mathbf{Q} = (1,1,2)$. By subtracting it from the measured $\mathrm{I}_{\mathrm{SF}}(\mathrm{T})$, the magnetic intensity can then be reported as, $\mathrm{I}_{\mathrm{mag}}(\mathrm{T}) = \mathrm{I}_{\mathrm{SF}}(\mathrm{T}) - \mathrm{I}_{\mathrm{SF}}^0(\mathrm{T}) = \mathrm{I}_{\mathrm{SF}}(\mathrm{T}) - \mathrm{I}_{\mathrm{NSF}}(\mathrm{T})/\mathrm{FR}_0(\mathrm{T})$. We applied this method in the data analysis presented in Fig. 3 of the main manuscript.

Note that, for a quantitative comparison of the magnetic intensities of both samples, all raw intensities have been background-subtracted, normalized to the same monitor counts and weighted by an estimated sample weight. The Supplementary Fig. 4 depicts scans of the nuclear Bragg peak intensity at $\mathbf{Q} = (1,1,2)$ along H in both samples. Due to a broader mosaicity of coaligned crystals, the peak width is broader in the doped sample. From Supplementary Fig. 4, one deduces the integrated intensity of the Bragg peak, which is in the doped sample $\sim$1.6 times smaller than in the pure sample. Taking that into account, one could compare the magnetic intensity of the observed hidden order around 240 K for both the pure and doped samples. We found a similar amount of the magnetic intensity in both samples as shown in Fig. 3b-c of the manuscript.

Supplementary Note 3

Relation between the hidden magnetic order and AFM order

The fact that we have ascribed the magnetic intensity at $\mathbf{Q} = (1, 1, 2)$ to a hidden magnetic order deserves additional comments. Briefly, the hidden order at $\mathbf{Q} = (1, 1, 2)$ cannot be described by any stacking of the planar pseudo-spin, $J_{\text{eff}} = 1/2$, antiferromagnetic (AFM) pattern. Below, we discuss different AFM models considered in the literature:

First, in the AFM $- + + -$ model (AF-I), the magnetic intensity is expected at some $\mathbf{Q} = (1, 1, L)$. However, it should be zero for L=2 because it does not preserve the body-centered symmetry of the unit cell, so $H + K + L$ should be odd or $L = 2n + 1$. As a matter of fact, a tiny magnetic intensity (about a hundred times weaker than at $\mathbf{Q} = (1, 0, 2)$) has been reported at $\mathbf{Q} = (1, 1, 1)$ in pure Sr_2IrO_4 [6]. That contribution is much weaker than at $\mathbf{Q} = (1, 0, L)$ because the magnetic intensity at $\mathbf{Q} = (1, 1, L)$ is proportional to the non-collinear pattern of the Ir moment. It is typically proportional to the square of the planar tilt angle of the IrO_6 octahedra, $\sim \theta^2$ with $\theta \simeq 11°$.

Second, in the AFM $+ + + +$ model (AF-II), a magnetic intensity at $\mathbf{Q} = (1, 1, 2)$ could exist in principle due to the ferromagnetic component. However, this interpretation can be excluded in both samples we have studied. First, in the pure sample, the ferromagnetic order is absent (see Fig.1 of the manuscript) [6,7] and can be only induced by an applied magnetic field of about 2000 Oe [3].

Second, under Rh substitution, such a ferromagnetic order indeed develops but only below $T_{\rm N}$ [8] as it results from the canting of the AFM order, clearly lower than $T_{\rm mag}$. We therefore rule out the weak ferromagnetism derived from the canted AFM order as a candidate to account for the observed magnetic scattering at $\mathbf{Q} = (1, 1, 2)$.

Third, in the hypothethic $- + -+$ model considered by Di Matteo and Norman [9], a magnetic intensity will be also present at some $(1, 1, L)$ positions. The structure factor for the $- + -+$ stacking is not zero only for even L as it keeps the body-centered symmetry of the unit-cell. Further, due to the glide plane, the structure factor is proportional to $\sin(\pi/2(H + L/2))$. Actually, it gives zero intensity at $(1, 1, 2)$ and then is not consistent with our finding. Further, such an AFM phase, $- + -+$, would also lead to magnetic contributions at $(1, 0, L)$ for odd L, which are not observed in any neutron diffraction reports [6,7] in pure $\rm Sr_2IrO_4$ as well as shown in the Fig. 2d of the manuscript.

Finally, one should stress that magnetic contribution from any of these AFM phases at $(1, 1, L)$ would be negligible as it is systematially proportional to $\sim \tan^2\theta$ with $\theta \simeq 11°$. Then, any effect around that $\mathbf{Q}$-position would be quantitatively too weak to be measured in our experiment. In absence of a planar tilt, it would even be strictly zero, although the signal for the LC phase will be still not zero.

Supplementary References

1. Fauqué, B. *et al.* Magnetic order in the pseudogap phase of high-T_c superconductors. *Phys. Rev. Lett.* **96**, 197001 (2006).

2. Bourges, P. & Sidis, Y. Novel magnetic order in the pseudogap state of high-T_c copper oxides superconductors. *C. R. Physique* **12**, 461–479 (2011).

3. Kim, B. *et al.* Phase-sensitive observation of a spin-orbital mott state in Sr_2IrO_4. *Science* **323**, 1329–1332 (2009).

4. Qi, T. *et al.* Spin-orbit tuned metal-insulator transitions in single-crystal $Sr_2Ir_{1-x}Rh_xO_4$ $(0 \leq x \leq 1)$. *Phys. Rev. B* **86**, 125105 (2012).

5. Ye, F. *et al.* Structure symmetry determination and magnetic evolution in $Sr_2Ir_{1-x}Rh_xO_4$. *Phys. Rev. B* **92**, 201112 (2015).

6. Ye, F. *et al.* Magnetic and crystal structures of Sr_2IrO_4: A neutron diffraction study. *Phys. Rev. B* **87**, 140406 (2013).

7. Dhital, C. *et al.* Neutron scattering study of correlated phase behavior in Sr_2IrO_4. *Phys. Rev. B* **87**, 144405 (2013).

8. Clancy, J. *et al.* Dilute magnetism and spin-orbital percolation effects in $Sr_2Ir_{1-x}Rh_xO_4$. *Phys. Rev. B* **89**, 054409 (2014).

9. Di Matteo, S. & Norman, M. R. Magnetic ground state of Sr_2IrO_4 and implications for second-harmonic generation. *Phys. Rev. B* **94**, 075148 (2016).
